# Health Tract, No. 223

**Published:** 1864-10

**Authors:** 


### ELEMENTS OF FOOD.

The ultimate ingredients of all food are carbon to warm, and nitrogen to make flesh. Some have no carbon, others no nitrogen, some have both in varying proportions, all have water or waste from five to ninety per cent. The table below is the result of the researches of the ablest chemists of the age. The amount of solid matter in an article of food, does not mean that amount of nutriment, for a portion of it may be woody fibre or waste, or lime, chalk, iron or other mineral. The cypher indicates that not one per cent of the element is found ; na : not ascertained ; blanks mean no published or reliable statements have been made. The more water, the more waste, for even woody fibre and iron have their essential uses in the system.

| In 100 parts of, there is per centage of | Solid matter. | Water. | Carbon. | Nitrogen. |
|---|---|---|---|---|
| Arabic gum | 88 | 12 | 36 | 0 |
| Artichokes | 28 | 80 | 9 | 0 |
| Apricots | 25 | 75 | | n.a. |
| Arrow root | 82 | 18 | 36 | n.a. |
| Almond oil | 100 | 0 | 77 | 0 |
| Butter | 83 | 17 | 66 | n.a. |
| Bread | 68 | 32 | 31 | n.a. |
| Beans | 87 | 14 | 38 | n.a. |
| Blood | 20 | 80 | 10 | 3 |
| Beef fresh | 25 | 75 | 10 | 8 |
| Beef tea | 2 | 98 | — | n a. |
| Cabbage | 8 | 92 | — | 0 |
| Carrots | 12 | 88 | — | 0 |
| Cherries | 25 | 75 | — | — |
| Cucumbers | 3 | 97 | — | — |
| Candy | 90 | 10 | 43 | 0 |
| Egg white, of | 20 | 80 | — | — |
| Egg yolk | 46 | 54 | — | — |
| Fish average | 20 | 80 | — | — |
| Figs | 84 | 16 | — | — |
| Gooseberries | 18 | 81 | — | — |
| Hogs lard | 100 | 0 | 79 | 0 |
| Isinglass | 92 | 7 | — | — |
| Leguminous seeds | 0 | 0 | 37 | — |
| Lentils | 84 | 16 | 37 | — |
| Manna | — | 40 | — | — |
| Mutton suet | 100 | — | 70 | 0 |
| Milk of cow | 13 | 87 | — | — |
| Milk of ass | 8 | 92 | — | — |
| Milk of goat | 13 | 86 | — | — |
| Olive oil | 100 | — | 77 | — |
| Oats | 79 | 21 | 40 | 2 |
| Oat meal | 93 | 7 | — | — |
| Oysters | 1s | 87 | 36 | — |
| Peas | 84 | 16 | — | — |
| Potatoes | 24 | 76 | 11 | — |
| Peaches | 20 | 80 | — | — |
| Pears | 16 | 84 | — | — |
| Poultry | 23 | 77 | — | — |
| Rye | 83 | 17 | 39 | 2 |
| Sugar average | — | — | 42 | 0 |
| Starch average | 84 | 16 | 36 | — |
| Wheat | 86 | 14 | 39 | 2 |

